# Supplementary material for: A kinase translocation reporter reveals real-time dynamics of ERK activity in Drosophila
Source: Biol Open. 2022 May 24;11(5):bio059364. doi: 10.1242/bio.059364 (PMC9167624; doi:10.1242/bio.059364)
Supplement: Supplementary information [file biolopen-11-059364-s1.pdf]

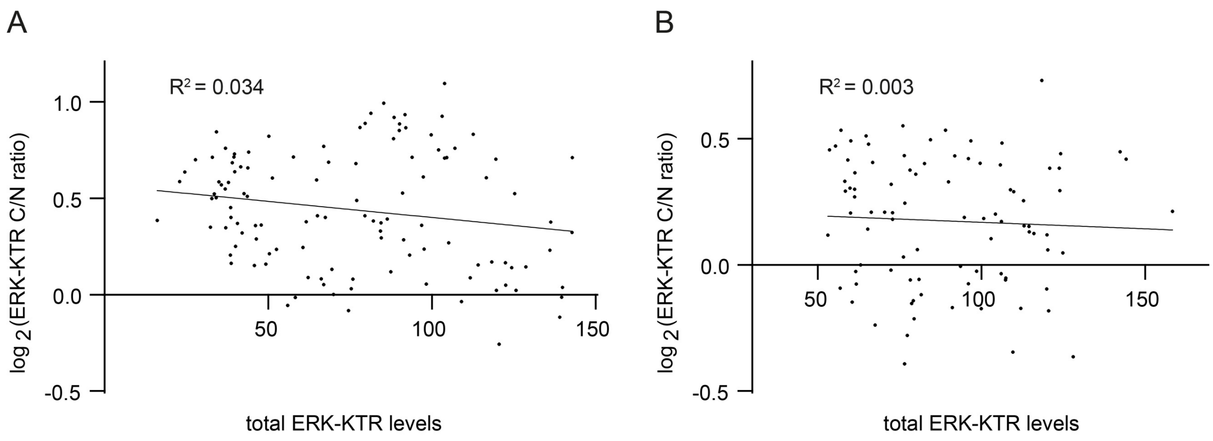

Fig. S1. The C/N ratio of ERK-KTR does not depend on total expression levels. Plots showing ERK-KTR C/N ratios against total expression in eye imaginal discs (A) and in follicle cells (B). Datasets were fitted with simple linear regressions. $R^2 = 0.034$ for eye discs and $R^2 = 0.003$ for ovarioles.

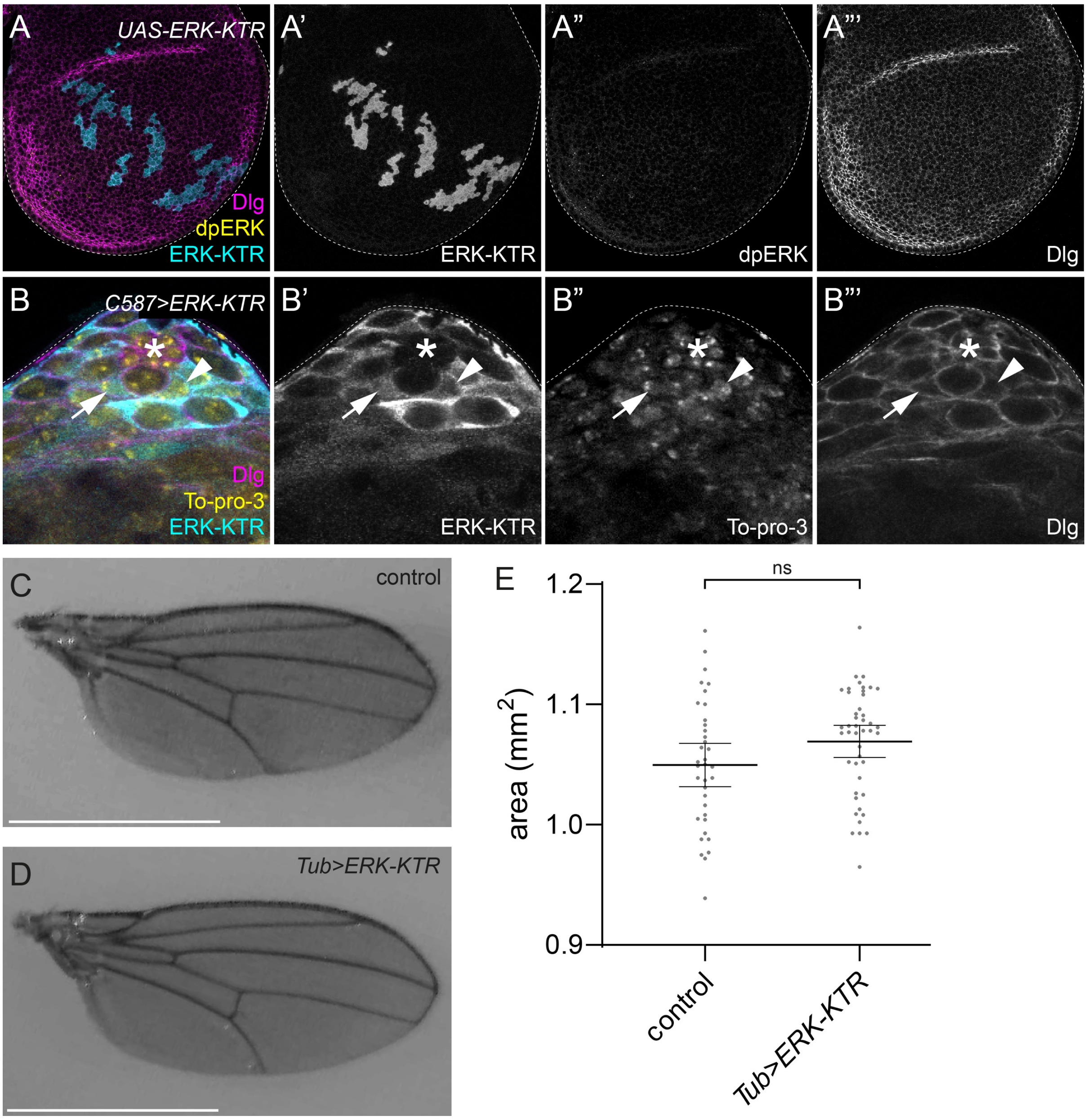

Fig. S2. ERK-KTR expression in clones, adult testes and driven by a ubiquitous driver. (A) Representative image of early 3rd instar larval wing imaginal disc with ERK-KTR-expressing clones (cyan, single channel A'), showing low, uniform dpERK (yellow, single channel A"). Dlg (magenta, single channel A"') labels cell outlines. (B) Somatic lineage-specific expression of ERK-KTR (cyan, single channel B') driven by *C587-Gal4* in the control adult testis. To-pro-3 (yellow, single channel B") labels cell nuclei, Dlg (magenta, single channel B"') labels cell outlines. Arrowhead indicates a cell in which ERK-KTR is excluded from the nucleus. Arrow indicates a cell with nuclear enrichment of ERK-KTR. (C,D) Representative images of adult wings from control flies (C) and flies ubiquitously expressing ERK-KTR driven by *Tub-Gal4* (D). The pattern of wing veins in ERK-KTR-expressing flies was indistinguishable from control flies (N = 36 wings for *Tub-Gal4* control, N = 45 wings for *Tub>ERK-KTR*). (E) Wing size was not significantly different between control and ERK-KTR-expressing flies (P<0.0743, Student's t-test). Bars show mean and 95% confidence interval. Scale bar = 10 µm.

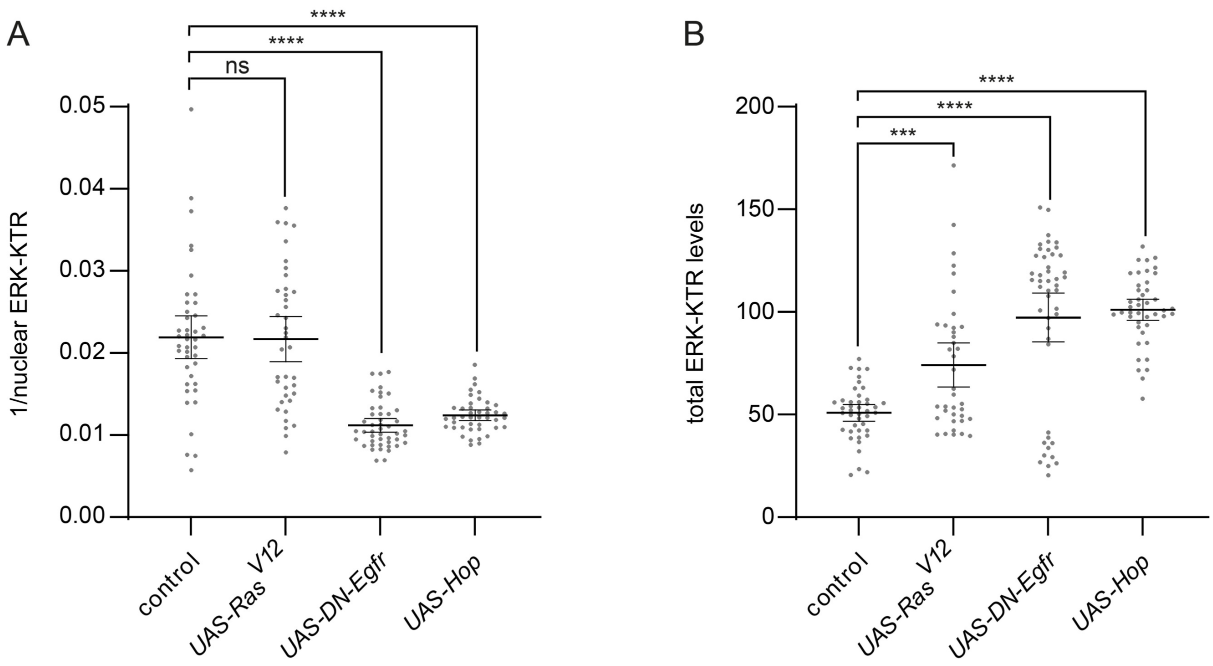

Fig. S3. Nuclear ERK-KTR intensity is an unreliable readout of ERK activity due to variable expression levels. (A) Plot showing the inverse of nuclear ERK-KTR levels from the clones shown in Fig. 2. DN-Egfr- and Hop-expressing clones had significantly increased nuclear intensity, while Ras^{V12}-expressing clones were not different from control (P=0.99968 for Ras^{V12}-expressing clones compared to control, P<0.0001 for DN-Egfr-expressing clones compared to control and for Hop-expressing clones compared to control). (B) Plot showing total ERK-KTR levels in clones of the indicated genotypes. All experimental clones had significantly different total levels from control (P=0.0003 for Ras^{V12}-expressing clones compared to control, P<0.0001 for DN-Egfr-expressing clones compared to control and for Hop-expressing clones compared to control), suggesting that differences in total expression prevent using measurements of nuclear levels as a readout for ERK activity. Lines show mean and 95% confidence interval. Statistical significance was determined using one-way ANOVA followed by Holm-Šidák's multiple comparisons test. ns = not significant.

Biology Open • Supplementary information

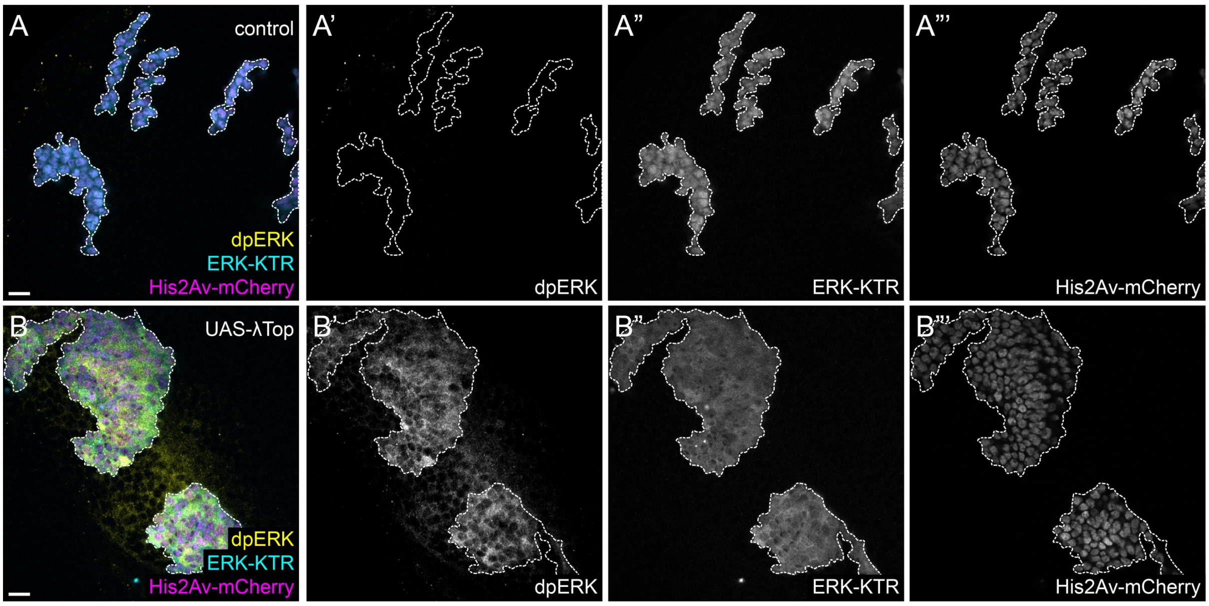

Fig. S4. ERK activity is increased in λTop-expressing clones. (A,B) Clones expressing ERK-nKTR labelled with an antibody against dpERK (yellow, A',B'). ERK-KTR is in cyan and His2Av-mCherry is in magenta. dpERK is undetectable in control clones (A) but highly upregulated in λTop-expressing clones (B). Dashed lines outline clones. Scale bar = 10 µm.

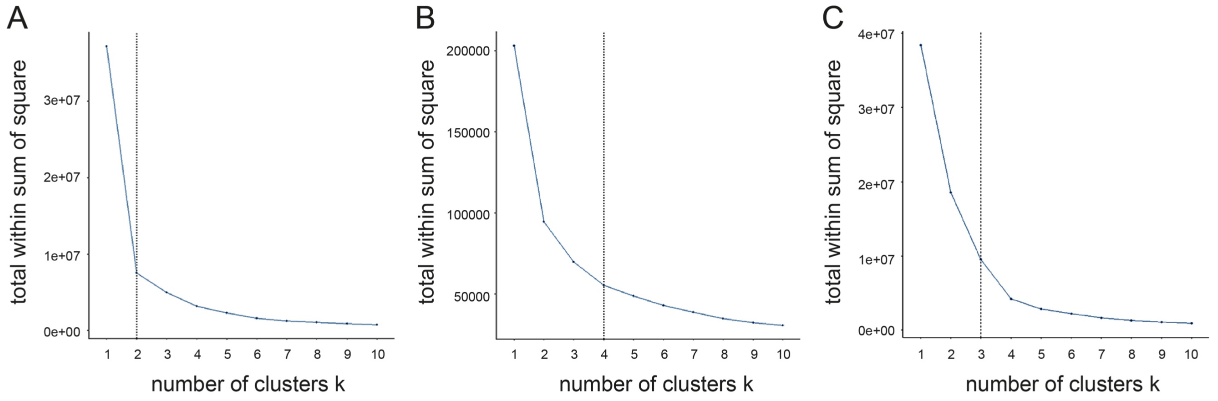

Fig. S5. Elbow plots used for determining the optimal number of clusters for Fig. 4D (A), Fig. 4G (B), and Fig. 5D (C).

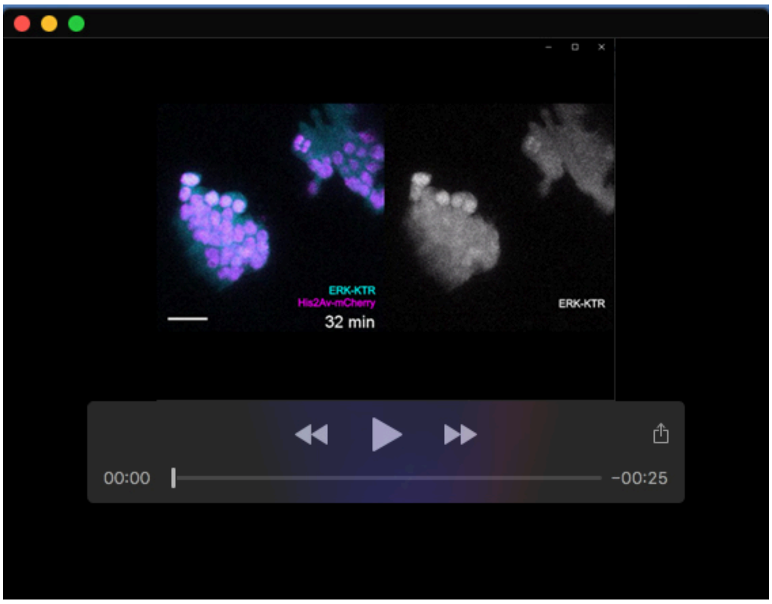

Movie 1. Time-lapse movie of a control flip-out clone expressing ERK-nKTR in the early third instar larval wing disc pouch. ERK-KTR is in cyan in the overlay and shown as a single channel in the right panel; His2Av is in magenta in the overlay. The image shown is a maximum projection of a Z stack. Timescale displayed in minutes. Scale bar = 10 µm.

Biology Open • Supplementary information

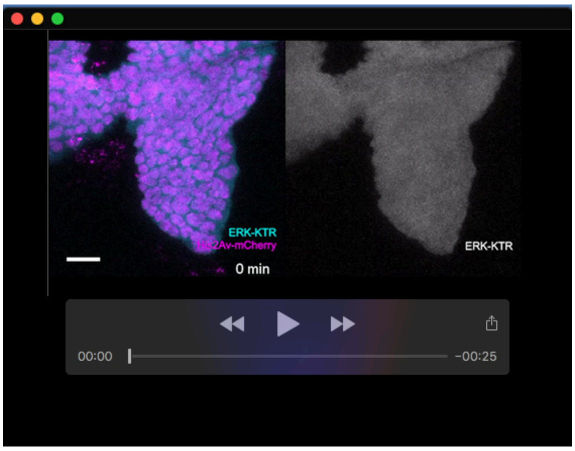

Movie 2. Time-lapse movie of a clone expressing ERK-nKTR together with λTop in the third instar larval wing disc pouch. ERK-KTR is in cyan in the overlay and shown as a single channel in the right panel; His2Av is in magenta in the overlay. The image shown is a maximum projection of a Z stack. Timescale displayed in minutes. Scale bar = 10 µm.

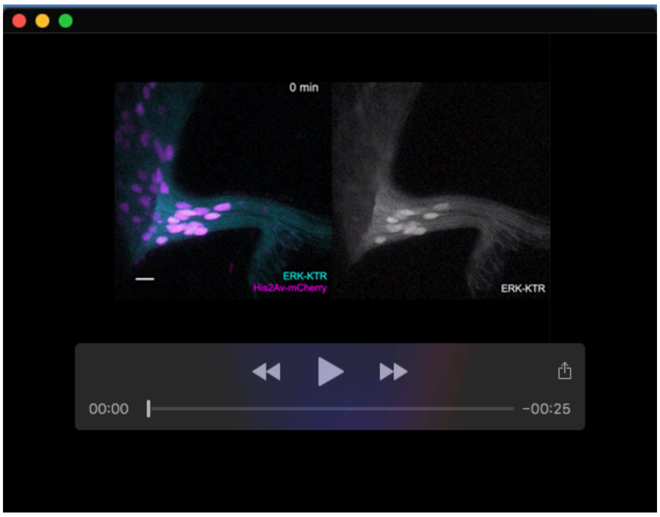

Movie 3. Time-lapse of an explanted visual system centred on the optic stalk in which ERK-nKTR was expressed using *R94A08-Gal4*. Explant is oriented as shown in the schematic in Fig. 5A, with the eye disc to the left and the optic lobe to the right. ERK-KTR is in cyan in the overlay and shown as a single channel in the right panel; His2Av is in magenta in the overlay. Arrowhead marks a nucleus with low ERK-KTR nuclear enrichment migrating towards the lamina; arrow points to a nucleus with highly nuclear-enriched ERK-KTR migrating towards the eye disc. The image shown is a maximum projection of a Z stack. Timescale displayed in minutes. Scale bar = 10 µm.

Biology Open • Supplementary information
